# Advances in enteric disease vaccines: from innovation to implementation

**DOI:** 10.1586/14760584.2014.882235

**Published:** 2014-02-05

**Authors:** Edward PK Parker

**Affiliations:** ^a^ Department of Infectious Disease Epidemiology, Imperial College London, Medical School Building, St Mary's Campus, Norfolk Place, London W2 1PG, UK

## Abstract

**The Seventh International Conference on Vaccines for Enteric Diseases** *Bangkok, Thailand, 6–8 November 2013*

Hosting 222 participants from more than 25 countries, the Seventh International Conference on Vaccines for Enteric Diseases (VED 2013) displayed the considerable progress that has been made in recent years towards unraveling the burden and etiology of enteric infections, alongside advances in the development, testing and implementation of vaccines that target them. The pervasive nature of enteric diseases, and the significant morbidity and mortality they account for, underscore the substantial public health benefits achievable through the use of enteric vaccines. A number of key talking points raised during the conference are discussed here, including early experiences with the use of double-mutant heat-labile enterotoxin as an antigen and adjuvant, progress and challenges associated with the implementation of oral cholera vaccines, and the issue of impaired rotavirus vaccine immunogenicity in lower-income countries.

## Burden & epidemiology of enteric diseases

Enteric diseases have long been recognized as a significant public health concern. However, recent years have seen a notable shift in our understanding of the extent and subtle nature of this burden. As highlighted by Myron M Levine (University of Maryland, Baltimore, MD, USA), the Global Enteric Multicenter Study (GEMS) – a project examining the burden and major etiologies of diarrhea in sub-Saharan Africa and south Asia – has emphasized the common occurrence of putative enteric pathogens not only among diarrheal cases, but also in nondiarrheal controls [1]. What, then, distinguishes the individuals who suffer (often severely) at the hands of a particular enteric infection from those who do not? Levine discussed the challenges posed by this question and noted that by estimating the attributable fraction of the overall diarrheal disease burden for each of a multitude of pathogens, it has been possible to shed light on the major players of diarrheal disease morbidity in infancy. In the first year of life, for instance, rotavirus was observed to account for the largest attributable fraction of moderate-to-severe diarrhea, followed by *Cryptosporidium*, enterotoxigenic *Escherichia coli* (ETEC) and *Shigella* [1].

Dennis Lang (National Institutes of Health, Bethesda, MD, USA) subsequently described the progress of the ongoing Malnutrition and Enteric Diseases (MAL-ED) project – a multicenter cohort study examining the relationship between malnutrition, enteric infections, vaccine performance and child development in eight low- and middle-income countries [2]. Like GEMS, the MAL-ED study's early findings have highlighted the frequent occurrence of enteric pathogens within both diarrheal and nondiarrheal stool samples. Lang also noted the frequent detection of multiple infections, with as many as seven putative pathogens observed per sample. By highlighting the pervasive and ‘often cryptic’ nature of enteric infections, the opening presentations by Levine and Lang laid down the gauntlet for the field of enteric vaccine research.

James Platts-Mills (University of Virginia, Charlottesville, VA, USA) followed these presentations by describing recent developments in the use of TaqMan^®^ array cards – a real-time quantitative PCR-based assay that enables the simultaneous detection of multiple enteric pathogens [3]. As emphasized by Platts-Mills, not all nucleic acid amplification is created equal: there is a balance between overdetection of pathogens by sensitive PCR-based techniques and the detection of clinically relevant infections. An important challenge, therefore, lies in determining this ‘sweet spot’ of pathogen detection. Given the prevalence of mixed infections highlighted by the GEMS and MAL-ED results, the array cards and similar technologies are poised to play a key role in enabling the complex etiologies of diarrheal disease to be unraveled.

## Vaccine developments: talking points from VED 2013

The conference's remaining program focused on recent developments in a range of enteric vaccines, with specific sessions devoted to ETEC, typhoid, *Shigella*, cholera, rotavirus, noroviruses and neglected enteric pathogens. Talks ranged in scope from the pathogenesis and genetic diversity of the infectious agents to progress in clinical trials and implementation of various vaccines. With as many as eight talks per session, this brief review will certainly fall short of capturing the range of advances and insights discussed. A selection of key talking points is outlined as follows.

### Double-mutant heat-labile enterotoxin: less is more?

As noted by Wilbur H Chen (University of Maryland, Baltimore, MD, USA), heat-labile enterotoxin (LT) derived from ETEC is an antigen of significant interest not only for its potential role within a complete ETEC vaccine, but also for its adjuvanticity. Chen described the development of double-mutant LT (dmLT) after a single-mutant variant proved too reactogenic in early trials. A Phase I trial was then described in which a single oral dose of dmLT was delivered to healthy adults at one of four levels (5, 25, 50 or 100 µg) [4]. The modified toxin was well tolerated at all doses and was immunogenic at both 50 and 100 µg. However, Chen noted that a consistent (albeit generally nonsignificant) trend toward a stronger immune response in 50 µg compared with 100 µg dmLT recipients was evident in a range of systemic and mucosal immune correlates, raising the possibility that the immunogenicity of dmLT may plateau with increasing dose.

The application of dmLT as an adjuvant was then described by Anna Lundgren (University of Gothenburg, Gothenburg, Sweden), who presented the results of a recent Phase I, double-blinded, placebo-controlled trial of a novel oral vaccine comprising the recombinant B subunit protein LCTB*A* alongside four inactivated ETEC strains overexpressing the colonization factors CS3, CS5, CS6 and CFA/I. Two doses of the vaccine were administered to healthy adults either alone, together with dmLT at a low or high dose, or with placebo. The vaccine was safe and well tolerated, and mucosal IgA antibody responses to all components of the vaccine were observed in approximately 75% of recipients. Notably, while no clear adjuvanticity was evident for dmLT overall, a trend toward enhanced immunogenicity was apparent in low-dose dmLT recipients. The findings were therefore consistent with the notion that the adjuvanticity of dmLT may be more potent at lower doses.

As noted by Clayton Harro (Johns Hopkins University, Baltimore, MD, USA), dmLT is the ‘new kid on the block’ of ETEC vaccine research. The coming years will undoubtedly see further insights into its potential value and optimal dose as an antigen and adjuvant.

### Experiences in cholera vaccine implementation

The conference's second day concluded with a special symposium on oral cholera vaccine (OCV) implementation. David Sack (Johns Hopkins University, Baltimore, MD, USA) introduced the session by highlighting recent landmarks for OCV, including the establishment of a vaccine stockpile by the WHO, the consideration of OCV for GAVI support and the delivery of more than 1.5 million doses of the licensed vaccines Dukoral^™^ and Shanchol^™^. Firdausi Qadri (International Centre for Diarrhoeal Disease Research, Dhaka, Bangladesh) then discussed a recent feasibility study for the use of Shanchol in Bangladesh [5]. The study involved delivery of more than 260,000 doses of OCV in urban Dhaka and further vaccinations in rural Keraniganj. While satisfactory coverage was obtained during the study, Qadri highlighted some of the operational challenges encountered during the scale-up of OCV delivery, including the substantial training requirements and (in particular) difficulties with cold chain management. These sentiments were echoed by Binod Sah (International Vaccine Institute, Seoul, Republic of Korea), who described a pilot study undertaken in the state of Odisha, India. In addition to challenges with cold chain maintenance, Sah discussed issues with vaccine packaging, as well as the less-than-pleasant taste and smell of the vaccine, which were among the key obstacles faced in reaching the desired coverage within the trial.

Subsequent presentations in the symposium highlighted the wide variety of settings in which OCV has been implemented, including the Mae La refugee camp in Thailand, urban and rural Haiti, and as part of Vietnam's national immunization program. Despite the challenges identified, the presentations emphasized the good acceptability of OCV and the potential to obtain adequate vaccine coverage in each setting. Dipika Sur (National Institute of Cholera and Enteric Diseases, Kolkata, India) also presented findings of a placebo-controlled, cluster-randomized trial of Shanchol carried out in Kolkata, India, in which two doses of the killed whole-cell vaccine were found to have a cumulative 5-year efficacy of 65% in preventing *Vibrio cholerae* serogroup O1 diarrhea severe enough to require treatment [6]. Overall, OCV implementation was highlighted as a feasible strategy to complement and enhance cholera prevention efforts.

### Determinants of rotavirus vaccine immunogenicity

A recurring theme in the session on rotavirus vaccines was the issue of impaired vaccine immunogenicity and efficacy in lower-income settings. Reasons for this trend remain unclear, though malnutrition, maternal antibodies, environmental enteropathy and interference by concurrent oral poliovirus vaccine delivery are suspected to play a role. S Asad Ali (Aga Khan University, Karachi, Pakistan) described a series of trials carried out in Karachi, Pakistan, to examine the possible influence of alternative vaccine delivery schedules (6 and 10 week, 10 and 14 week or 6, 10 and 14 week delivery) and temporary withholding of breastfeeding on seroconversion rates following Rotarix^®^ administration. No significant differences in seroconversion rates or antirotavirus antibody titers were observed for the different schedules, while withholding breastfeeding was not associated with a boost in vaccine response. Similarly, the temporary withholding of breastfeeding had no impact on seroconversion rates in a trial of Rotarix recently conducted in Bangladesh (K Zaman, International Centre for Diarrhoeal Disease Research, Dhaka, Bangladesh).

Jacob John (Christian Medical College, Vellore, India) then described a trial conducted in Vellore, India, in which the effects of zinc and/or probiotic supplementation on seroconversion following two doses of Rotarix were examined. John drew attention to the higher-than-expected baseline rotavirus seropositivity observed in the study, alongside the poorer-than-expected vaccine response. Overall, zinc supplementation had no impact on rotavirus seroconversion, while probiotic supplementation was associated with an increase in seroconversion, albeit nonsignificant. The potential impact of zinc or probiotic supplementation on rotavirus vaccine efficacy remains uncertain.

Together, these and other presentations in the session highlighted the uncertainties that remain in our understanding of the determinants of rotavirus vaccine immunogenicity, and the further work that will be required to resolve the factors responsible for the impaired performance of the vaccine in lower-income settings.

## The road ahead

Following a plenary presentation by John Mekalanos (Harvard University, Cambridge, MA, USA) highlighting several aspects of bacterial pathogenesis that might be harnessed in the next generation of enteric vaccines (e.g., the type VI secretion system), Thomas Brewer (Bill & Melinda Gates Foundation, Seattle, WA, USA) closed the conference by highlighting the huge variety and depth of materials presented. Brewer drew attention to the key milestones achieved in recent years, including the invaluable insights gained from GEMS, significant progress in the implementation of OCV and a growing appreciation of the ‘multiple pathogen concept’ in our consideration of enteric infections.

In a field noted by Brewer to be ‘growing at a fairly exponential rate’, these advances form a firm platform on which to better tackle the considerable morbidity and mortality attributable to enteric diseases.
